# Early Compressive Deformation of Closed-Cell Aluminum Foam Based on a Three-Dimensional Realistic Structure

**DOI:** 10.3390/ma12111792

**Published:** 2019-06-03

**Authors:** Xiong Wan, Kai Zhu, Yanjin Xu, Baoshuai Han, Tao Jing

**Affiliations:** 1 Key Laboratory for Advanced Materials Processing Technology, Ministry of Education, School of Materials Science and Engineering, Tsinghua University, Beijing 100084, China; wanx15@outlook.com; 2 State Key Laboratory of Non-Ferrous Metals and Processes, General Research Institute for Nonferrous Metals, Beijing 100088, China; zhu_thirteen@foxmail.com; 3 AVIC Manufacturing Technology Institute, Beijing 100024, China; hbshit@126.com

**Keywords:** early compressive deformation, closed-cell aluminum foam, plastic strain band, X-ray computed tomography

## Abstract

It is well-known that cell morphology plays a vital role in the mechanical properties of the closed-cell aluminum foam. In this work, a three-dimensional (3D) realistic structure was obtained by using the synchrotron X-ray micro-tomography technique and then translated into a numerical model for a further finite-element simulation. In order to investigate the early compressive deformation in the closed-cell aluminum foam, we chose three different strain levels, namely, 0.2% (initiation of plastic strain), 2.8% (propagation of plastic strain band), and 6% (formation of collapse band) to discuss the evolution forms of plastic strain concentration by simulation. We found that the curvature, anisotropy, and distribution of cell volume of adjacent cells played a vital role in the initiation of plastic strain. Furthermore, the phenomenon that plastic strain band propagated along the direction aligned 45° with respect to the orientation of the compression was also investigated in the propagation of the plastic strain band and formation of the collapse band. Finally, the comparison between experimental results and simulation results was performed to illustrate the early location of these three different levels in the whole compressive deformation.

## 1. Introduction

Lightweight closed-cell aluminum foam configurations possess not only good specific strength and stiffness but also outstanding energy absorption and wave attenuation abilities when subjected to an external load [1,2,3,4,5,6]. These excellent qualities make them ideal candidates in military applications, aerospace, and civil industry [7,8,9]. It is well known that the mechanical properties of the closed-cell foam depend on their micro- or mesoscale cellular structure. Hence, a lot of experimental and numerical investigations have been carried out to study the deformation behavior of large-scale strain concentrated regions when subjected to different kinds of loading states [2,4,10,11,12,13,14]. 

In previous experimental works, it was already demonstrated that cell morphologies and structures play an important role in the initiation and propagation of the deformation behavior of the closed-cell aluminum foam [6,15,16,17,18]. These results indicated that the collapsed band always initiate from cells with their longest semi-axis perpendicular to the loading direction. However, all topographies were only observed during interruptions in different compression tests. That is, until now, the evolution of strain distribution during compressive tests has been hard to check.

Concerning the numerical simulations, some previously constructed models consisting of periodical and regular unit-cells could not reflect the effect of various and actual cell structures on the material properties. For example, Nammi et al. used a numerical model consisting of a series of repeating tetrahedral unit-cells to represent the intricate structure of the closed-cell aluminum foam and then simulated the stiffness and mechanical response of this model under uniaxial quasi-static loading [19]. Some results were only given in a 2D perspective because of the opaqueness of metallic materials restricting the observation extents of the traditional methods [2,20]. Recently, Saadatfar et al. adopted X-ray computed micro-tomography to gain access into the 3D structure of aluminum foam and investigated its mechanical response before and after deformation [6]. Under the quasi-static uniaxial compression, it was found that the regions with high degrees of local anisotropy belonging to the under-formed foam are likely to induce collapse bands [6]. However, in that work, some questions still need to be further investigated and discussed, i.e., how stress concentration initiates in foam cells, how the plastic strain band propagates, and how collapse bands forms.

To clarify these questions, we adopted synchrotron X-ray micro-tomography to reveal and characterize real 3D structures of the closed-cell aluminum foam in this work. The extracted data from the radiographs were post-processed, and then these data were reconstructed and used as the input model of ABAQUS. Therefore, in this work, we could give numerically full-scale structure information about the deformation procedure of closed-cell aluminum foam with the help of synchrotron X-ray micro-tomography. In addition to the numerical simulations, experimentally quasi-static uniaxial compressive tests were also conducted to invalidate the results of the numerical simulations.

## 2. Materials and Methods

### 2.1. Specimens Preparation

**Figure 1:**
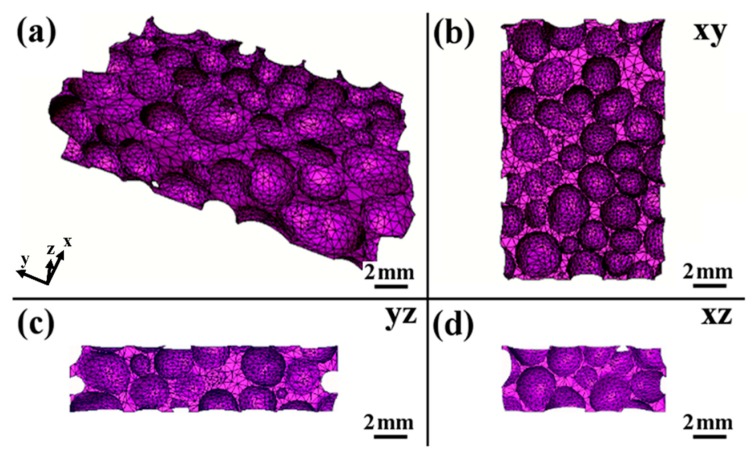
Compatible 3D finite-element meshes obtained by using the Amira software: (**a**) the whole mesh was shown in 3D dimensional diagram; (**b**–**d**) the cross-sectional diagrams from three mutually perpendicular planes.

In this research, the examined closed-cell aluminum foam was fabricated via a melt route by adding 2.0% CaH_2_ via the melt foaming process at 660 °C; more details of the fabrication process have been presented elsewhere [21]. A small sample with a dimension of 4 mm × 10 mm × 15 mm, shown in Figure 1, was cut from a large aluminum foam plate by electro-discharge machining. The density of the closed-cell aluminum foam was calculated to be 0.54 g/cm^3^ (weight divided by the volume of the sample), and the average diameter of this closed-cell aluminum foam was about 2 mm.

### 2.2. Synchrotron Radiation X-ray Tomography

The beamline BL13W1 in Shanghai Synchrotron Radiation Facility (SSRF) was applied to gain access into the internal real three-dimensional (3D) structure of the closed-cell aluminum foam. In consideration of the resolution and the dimension of the specimen, we chose the detector with a pixel size of 9 μm. An appropriate silicon crystal plate was used to gain the tunable monochromatic X-ray energy of 24 keV, which could be absorbed partially by the aluminum foam. The distance between the specimens and the detector was adjusted several times and finally determined to be 8 mm, enhancing the edge detection and ensuring the whole specimen was located in the field of X-ray. A YAG:Ge scintillator screen assembled in the front of the detector was designed to convert the X-rays to visible light. Then, the photoelectric conversion of visible light was conducted by an Opqitue Peter CCD (Charge Coupled Device) camera (the size of the field was 2048 × 2048 pixels). The specimen was then fastened in the center of the sample stage, which could rotate together with the attached specimen on its upper surface around its own central axis. Totally, 900 projections were collected during the *pre*-determined 180° rotation with an exposure time of 0.2 s for each step (0.2°).

### 2.3. Real 3D Model Reconstruction

After obtaining the whole set of sequential projections, a free software PITRE (Shanghai Synchrotron Radiation Facility, Shanghai, China) was used to process the phase-sensitive X-ray image and perform tomography reconstruction, acquiring a series of computed tomography (CT) images. Then, noise reduction and binarization of that tomography were conducted in ImageJ, a free image processing software, to get eight-bit bitmaps which contained the structural information of the cells in the aluminum foam specimens. Next, a series of bitmaps were used to generate the real 3D model of the specimens comprising the computed surfaces. Subsequently, after the smoothing and optimization of these surfaces, the surface meshes generation process was performed, after which a relative tetrahedral mesh with high quality was obtained on the base of the surface mesh, as shown in Figure 1.

### 2.4. Simulation of Compression

**Figure 2:**
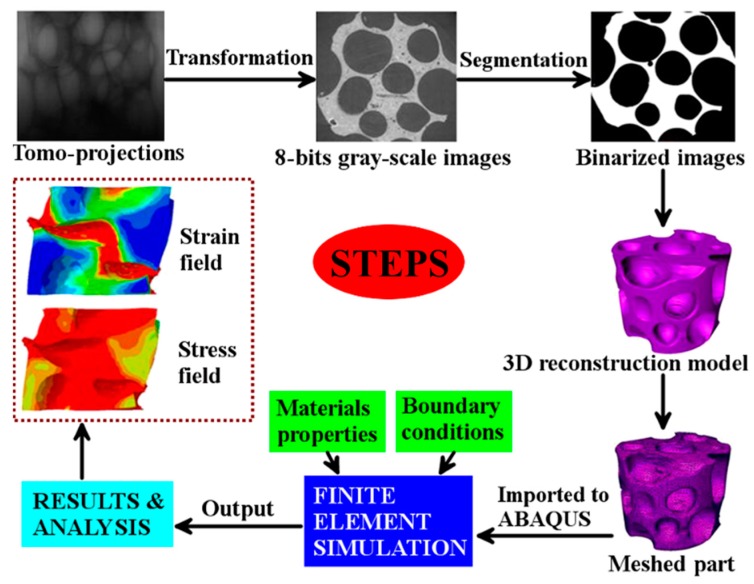
Detailed procedure of model reconstruction and simulation.

A finite-element model (FEM) was employed to simulate the quasi-static compression using the commercial code ABAQUS/Explicit to analyze the distribution evolution process of stress and strain during the externally uniaxial compressive load. Before the experiment of simulation of compression, the intrinsic mechanical properties of the raw aluminum material were evaluated by additional standard testing experiments. The Young’s modulus, Poisson’s ratio, and yield stress of the base material (pure aluminum) were measured to be 72.0 ± 0.8 GPa, 0.33 ± 0.02, and 35 ± 2 MPa, respectively. The quadratic tetrahedral elements with 10 nodes (marked “C3D10M” in ABAQUS) were applied for the closed-cell aluminum foam. Furthermore, the platens contacting the specimens in the compression experiment were treated as analytical rigid parts. Meanwhile, a frictionless contact interface between the specimen and the rigid parts was applied to simulate the experimental boundary conditions. Additionally, it should be noted that no fracture criterion was utilized during this simulation process because we only focused on the initial plastic deformation of the partial cells in the aluminum foam specimens, wherein the fracture of the cell walls could be ignored. Besides, for the simplification of the simulation process, imperfections of the cell wall microstructure were also not introduced in our simulation, such as corrugation, bowed walls, damaged wall, and non-uniform thickness. Figure 2 is presented to describe the above whole process including the experimental observations and numerical simulation process.

### 2.5. Compression Experiment

The specimens were cut from a large aluminum foam plate by the electro-discharge machining (EDM) to minimize the undesirable damage of local cell walls. In order to measure the Young’s modulus of the closed-cell aluminum employed in this work more accurately, five standard rectangular specimens with dimensions of 30 mm × 50 mm × 30 mm were prepared for the compressive test. Moreover, all the tests were conducted under displacement control module with a cross-head speed of 0.5 mm·min^−1^.

## 3. Results and Discussion

### 3.1. Simulation Results and Initial Deformation Behavior

**Figure 3:**
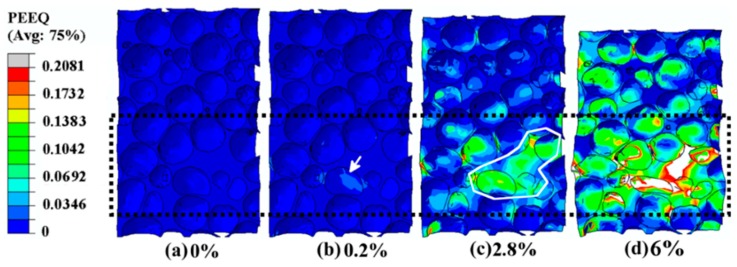
Computed strain distribution in a specimen at different strain deformation levels. PEEQ (indicates the equivalent plastic strain in ABAQUS): initiation and evolution of the equivalent plastic strain. (**a**) the strain distribution of the specimen before loading, showing no strain concentration; (**b**) the strain distribution of the specimen at its overall strain of 0.2%, showing the initiation of the plastic strain; (**c**) the strain distribution of the specimen at its overall strain of 2.8%, showing the propagation of the plastic strain band; (**d**) the strain distribution of the specimen at its overall strain of 6%, showing the formation of the collapse band in the specimen.

The initial deformation behavior during the simulation of the compression experiment was investigated using the initiation and evolution of the equivalent plastic strain (indicated by PEEQ in ABAQUS) in the longitudinal section of the aluminum foam specimens along the orientation of compression, as shown in Figure 3. The original and deformed simulation results of the specimens subjected to four different strain levels of 0%, 0.2%, 2.8%, and 6% werea chosen to explain the evolution of PEEQ in the process of cos mpression, shown in Figure 3a–d; the corresponding stress–strain curve are discussed in Section 3.2. 

#### 3.1.1. Initiation of Plastic Strain in the Simulation Results

Before loading, PEEQ field exhibited a uniform distribution, and no strain concentration could be found in the strain contour figure, as shown in Figure 3a. Note that the compression speed of the rigid platen in the simulation was set to 0.0025 mm·s^−1^, ensuring to record the initial strain information exhaustively. The strain rate implemented in compression simulation was set to be equal to that in the practical compression experiment, namely, 1.67 × 10^−4^·s^−1^.

**Figure 4:**
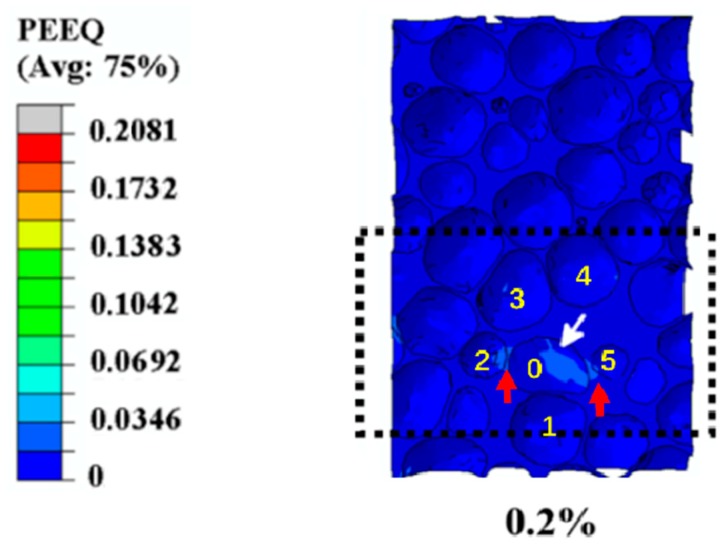
Illustration of the initial plastic strain in a large elliptical cells at strain level 0.2%.

Subsequently, as the load rose further till the overall strain (the displacement of the rigid platen divided by the original length of the specimen along the orientation of compression) of the specimen reached 0.2%, the equivalent plastic strain concentration formed at the large cell and its cell walls, which is marked by the white arrows in Figure 4. Obviously, it can be seen from Figure 4 that the initial strain concentration focused at the elliptical cells with relatively higher anisotropy and curvature, which is consistent with the results of a previous work [6]. Furthermore, there were five cells (marked “1”, “2”, “3”, “4”, and “5”) around the central strained elliptical cell (marked “0”), as shown in Figure 4. The cell walls between the central strained elliptical cell and two smaller cells were subjected to greater plastic deformation, marked by the red arrows in Figure 4. Ref. [22] points out that smaller cells around the central strained elliptical cell are more susceptible to deformation and suffer from the higher strain than the larger adjacent cells, which is in good agreement with our experimental results. 

This phenomenon suggests that the distribution of the cell volume of smaller adjacent neighbors plays a vital role in the initial plastic deformation [22]. If the accurate area and the basic features of the initial plastic strain can be affirmed, a closed-cell aluminum foam with higher stiffness and strength can be fabricated. 

#### 3.1.2. Propagation of the Plastic Strain Band in Simulation Results

**Figure 5:**
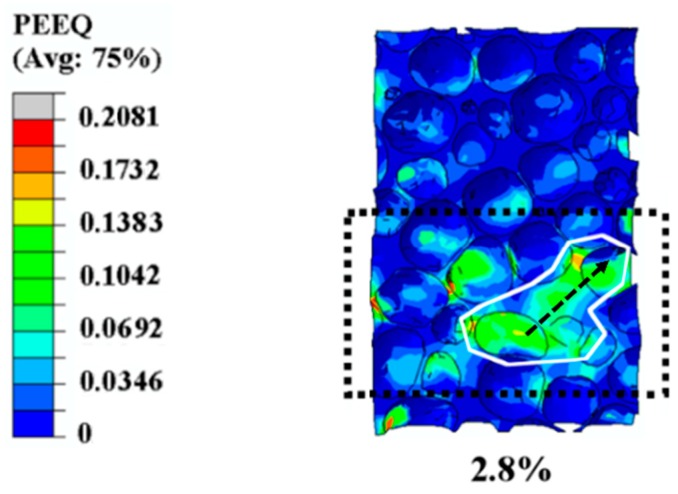
Illustration of the propagation of the plastic strain band at strain level 2.8%.

As the load increases gradually, the plastic strain concentration will propagate from the initial plastic deformation region to its adjacent area, which is shown in Figure 5. The orientation of the main propagation is marked by the black dotted arrow in Figure 5. It was found that the strain level within this deformation area was about four times larger than that in other sites, as well as one order of magnitude larger than the overall applied strain. Furthermore, this deformation band was obviously aligned 45° with respect to the orientation of the compression, which was in the direction of the maximum shear strain. This phenomenon is well in agreement with the results of previous research [1]. Besides, the local deformation bands, circled by a white line, exhibited the width of about 1–1.5 cell diameters, as was also reported in the Ref. [1].

#### 3.1.3. Formation of the Collapse Band in Simulation Results

**Figure 6:**
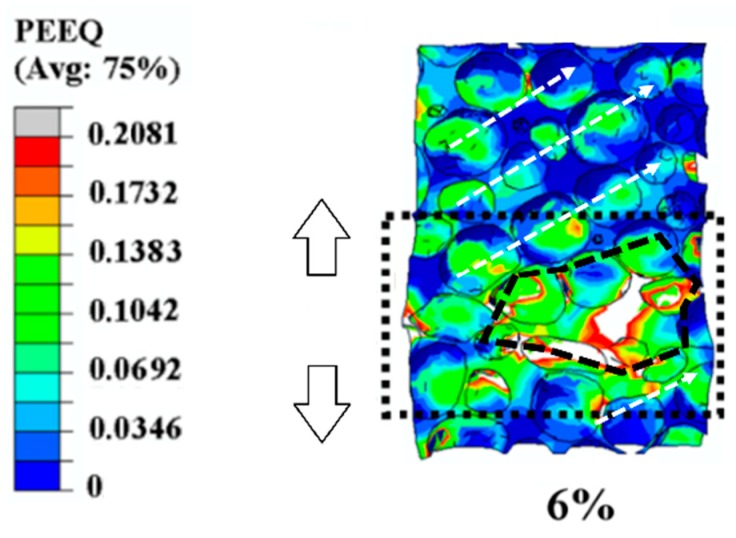
Illustration of the collapse band and the path of plastic strain concentration at strain level 6%. The up and down arrows on the left of the contour figure represent the overall propagation of the plastic deformation range.

When the overall applied strain reached 6%, an apparent collapse band formed due to excessive buckling by the increasing external load, and the width of the deformation region enlarged to 2–3 cell diameters, to penetrate through the whole thickness of the specimen, marked by the white dotted line in Figure 6, which is consistent with previous studies [19,23]. Additionally, cooperative deformation of the cells surrounding the collapse band was found; cooperative deformation in the aluminum foam specimen subjected the adjacent cells to plastic strain, which was shown by the enlargement of the plastic deformation range. The overall orientation of the propagation is marked by the left white arrows in the Figure 6. From Figure 6, we can see that the plastic strain range extended to the whole specimen before the collapse band spread through the whole width of the specimen, perpendicular to the loading direction, showing its excellent ability to disperse the external load and reducing the damage of the concentrated load. When the plastic strain propagated to the less deformed or no deformed areas gradually, the initial deformation behavior and the propagation of the plastic strain band were expected to evolve, as described in Section 3.1.1 and Section 3.1.1, respectively. For instance, the white dotted arrows shown in Figure 6 represent the plastic strain bands aligned 45° with respect to the loading direction, which is consistent with the description of plastic strain bands in Section 3.1.1 [1].

Most important, from a general survey of this deformation process, it was found that the initial strain concentration sites were localized in a cell (marked by a white arrow in Figure 4), which possessed a relatively irregular structure with higher anisotropy than other cells with equiaxed morphologies [2,4,6]. Specifically, there was a negative curvature resulting from the wavy membrane of the cell wall, marked in Figure 4, which was the important reason for the initial plastic strain according to the Ref. [2], which reports that high curved cell walls have a detrimental influence on the yield strength. Besides, Ref. [2,6] demonstrates that equiaxed cells begin to deform into elliptical morphologies, and the orientation of the longest axis of these elliptical cells tends to lie in planes perpendicular to the compressive loading direction, which is in agreement with our simulation results marked by the black dotted rectangular in Figure 3. Subsequently, this area and its surrounding area act as the most susceptible strain concentration area, and from here, the plastic strain band and collapse band form and expand outward successively [2,24]. 

### 3.2. Comparison of the Compression Test Results between Simulation and Experiment

**Figure 7:**
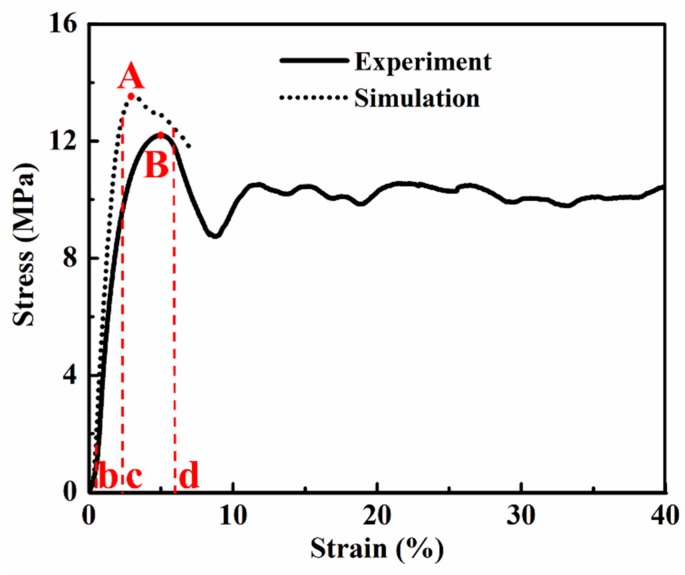
Compressive stress vs strain curves obtained from the experiment and the simulation. In this Figure, point A and point B represent the maximum stress values in experimental results and simulation results respectively, point b, c, and d represent three different strain values (0.2%, 2.8%, and 6%), corresponding to the strain contours in Figure 3b–d.

Typical experimental and calculated compressive stress–strain curves in our research are shown in Figure 7. When the aluminum foam was under compressive load, the stress–strain curve of the aluminum foam could be divided into three different stages [25,26]: a linear elastic deformation stage, a plastic deformation and pore collapse stage, and a densification stage. In the first stage, the foam deformed linearly and elastically, and the deformation was shown to be related to the elastic bending of the cell was in foam materials. Then, the foam deformed with almost constant stress, indicated by a plateau of deformation. Finally, the foam densified, and the stress increased rapidly again. In our study, we only focused on the early deformation of the foam, and the stress–strain curves are shown in Figure 7. 

In Figure 7, both curves show the intact linear elastic deformation stage and the partial stage of plateau deformation, where three different deformation levels, discussed above in Section 3.1.1, Section 3.1.2 and Section 3.1.3, occur successively, marked “b”, “c”, and “d”, corresponding to the strain contours in Figure 3b–d. Maximum stress points (marked “A” and “B” for simulation curve and experiment curve, respectively) are shown in the two curves in Figure 7, representing the onset of global collapse, followed by a load softening region and a “plateau” characterized by serration. These observations were also reported in Ref. [23,27].

The slope of the compressive stress–strain curves from the experimental results indicated that the elastic modulus of this closed-cell aluminum foam was 0.43 GPa. This value was supported by the general formula proposed by Simone and Gibson, that is, the relationship between the relative density and the relative Young’s modulus of closed-cell aluminum foam can be described as follows [9,17,28]:

(1) $$\frac{E^{*}}{E_{s}}=0.32{\left(\frac{\rho^{*}}{\rho_{s}}\right)}^{2}+0.32\left(\frac{\rho^{*}}{\rho_{s}}\right)$$

where E* is the Young’s modulus of the foam material, *E_s_* is the Young’s modulus of the master aluminum. Here, the density *ρ_s_* and the elastic modulus *E_s_* of the cell wall solid were taken as 2.7 g/cm^3^ and 72.0 GPa, respectively. Through calculation, it was found that the theoretical Young’s modulus of this foam material was about 0.44 GPa. Therefore, it could be observed that the experimental result was nearly consistent with that of the theoretical calculation but slightly lower than that of the numerical simulation (about 0.5 GPa). The difference of the elastic *E_s_* between the simulation results and the experimental results can be ascribed to the idealization of the foam structure during 3D model reconstruction, eliminating the imperfections (such as corrugations, damaged walls, bowed walls, and not uniform thickness [29]) which existed in the compression experiment, and the simplification of the simulation (for instance, fracture behavior of the cell walls were ignored). 

In Figure 7, the curve of the simulation results matches well with that of the experimental results, especially for the initial linear-elastic stage. Therefore, in the stage of initial plastic strain (0.2%) (discussed in Section 3.1.1, corresponding to Figure 3b and to the letter “b” in Figure 7), the simulation results reflected the actual deformation accurately, owing to the small deformation. Subsequently, when the deformation reached the level of propagation of the plastic strain band, the overall strain 2.8% (discussed in Section 3.1.2, corresponding to Figure 3c and to the letter “c” in Figure 7) was located left of point “A”, demonstrating that plastic strain formed before global collapse. Finally, another strain level (6%, discussed in Section 3.1.3, corresponding to Figure 3d and to the letter “d” in Figure 7) was located right of point “A”, which was chosen to represent the formation and evolution of the collapse band. Apparent differences were found between the simulation results and the experimental results in the latter two strain levels (2.8% and 6%), resulting from the idealization assumed in the simulation and the complexity of the experiment.

## 4. Conclusions

In this work, a finite-element model based on the real 3D microscopic structure of the closed-cell aluminum foam was successfully established using synchrotron X-ray micro-tomography and real 3D model reconstruction to investigate the evolution of plastic strain during early compressive deformation. Several conclusions can be drawn from this work, as follows:

1. The whole process of obtaining the finite-element model based on the real 3D structure was discussed using the synchrotron radiation X-ray tomography and 3D model reconstruction.
2. Different strain levels (0.2%, 2.8%, and 6%) were chosen to discuss the evolution of plastic strain in early compressive deformation. The curvature, anisotropy, and distribution of the volume around cells were found to be important for the initiation of the plastic strain at the lower overall strain level (0.2%). Furthermore, the plastic strain band propagated along the direction aligned 45° with respect to the loading direction, and the width of the plastic strain band increased gradually till the formation of the collapse band in the other two strain levels (2.8% and 6%, corresponding to the levels of propagation of the plastic strain band and formation of the collapse band).
3. The numerical and experimental results matched well. Meanwhile, three different strain levels were further demonstrated to exist in the early compressive deformation stage in the stress–strain curves, establishing a correlation between the evolution of the plastic strain among the cells and their global compressive deformation behavior.

More importantly, our research is based on a 3D realistic structure of closed-cell aluminum foam to validate some results proposed by previous reports based on 2D investigations of the evolution forms of plastic strain in early compressive deformation by simulation.
